# Conventional and Novel Strategies for Cellulose Isolation from Nut Shells: A Review

**DOI:** 10.3390/molecules30122486

**Published:** 2025-06-06

**Authors:** Andrea Están, Mónica Umaña, Valeria S. Eim, Gabriela Clemente, Susana Simal

**Affiliations:** 1 Department of Chemistry, University of the Balearic Islands, Ctra. Valldemossa, km. 7.5, 07122 Palma de Mallorca, Spain; andrea.estan@uib.cat (A.E.); monica.umana@uib.es (M.U.); valeria.eim@uib.es (V.S.E.); 2 Analysis and Simulation of Agro-Food Processes Group, University Institute of Food Engineering, FoodUPV, Universitat Politècnica de València, Camino de Vera s/n, 46022 Valencia, Spain; gclemen@tal.upv.es

**Keywords:** lignocellulose, nut shells, nut by-products, cellulose, lignin, cellulose extraction, pre-treatment

## Abstract

Nut by-products, particularly shells, are a globally abundant agricultural residue. Their widespread accumulation poses a serious environmental challenge. However, nut shells are of great interest due to their inherent lignocellulosic composition. For instance, they are rich in cellulose, a high-value biopolymer widely used in the production of bio-based materials. Therefore, this review critically analyses conventional and novel pre-treatment strategies for the extraction of cellulose from nut shells, emphasising the importance of optimising valorisation routes to minimise ecological impact. Various techniques—ranging from alkaline treatments to emerging approaches such as deep eutectic solvents and hydrothermal methods—have been examined and compared. The findings in cellulose purification through different strategies reveal that, while some methods are promising, others remain underexplored. Emphasis is placed on the necessity of comprehending the specific structural and chemical characteristics of each type of nut shell; as such, knowledge is fundamental to understanding the efficiency of the applied methods. This review highlights the growing interest in the valorisation of nut shell by-products as promising lignocellulosic resources of significant utility. Therefore, it also reveals the need for further research, focusing on process scalability, cost-efficiency, and environmental impact. Advancing in these areas is essential to enable the transition of nut shells from waste to a highly valuable resource.

## 1. Introduction

Nuts (almond, walnut, cashew, hazelnut, and macadamia, among the most popular ones) [1] have been an essential part of human diets for centuries, valued for their nutritional richness, long shelf life, and versatility in culinary traditions across cultures [2]. Their consumption dates back to ancient civilisations, used for food but also recognised for their inherent medicinal and functional properties [3].

Over time, the rise of highly processed snack foods led to a change in how nuts were consumed. However, in recent decades, growing awareness of the impact of diet on health and well-being has brought a renewed interest in nuts, as a part of the mindset change towards healthier eating habits [4]. Therefore, nuts have gained a path into being consumed for their natural form, offering a healthier, more nutrient alternative to processed snacks and encouraging a return to whole food ingredients that align with a modern wellness life. Studies have proven that the intake of nuts is inversely related to cardiovascular diseases or cancer [5], in contrast to processed snacks that can contribute to long-term health issues [6]. Moreover, their versatility and natural taste make them suitable for a variety of consumption methods, whether eaten alone (fresh or roasted), combined with other ingredients, or incorporated into different culinary preparations. Nuts have an interesting nutritional profile, rich in micronutrients [7] (e.g., folic acid, vitamin B6, calcium, magnesium), bioactive compounds [8,9] (e.g., unsaturated fatty acids, phospholipids, terpenoids, tocopherols), phytochemicals [10,11] (e.g., phenolic acids, flavonoids, tannins, alkaloids), and polysaccharides [12].

Moreover, beyond their role in human nutrition, nuts have also gained importance in other industries such as the cosmetic sector [13]. The rising demand for plant-based and naturally derived ingredients such as oils has expanded the use of nut-based formulations in skincare, haircare, and nutraceutical products, further increasing their economic and industrial significance [13,14].

To ensure successful germination in nature, these crops evolved to protect their fruits or seeds by different mechanisms such as walls and seed coats [15], developing multiple layers that separate the kernel from the outside, shielding its delicate inner part from external threats [16]. However, these protective layers are not edible and therefore often discarded, although they contain a significant potential for upcycling due to their interesting composition: the nut husks, shells, skins, or even pruning fibers have a lignocellulosic matrix rich in polysaccharides, bioactive compounds, fibers, proteins, and vitamins [17,18].

These lignocellulosic by-products, primarily composed of cellulose, hemicellulose, and lignin, represent a valuable resource in the nut industry. Cellulose, the most abundant polysaccharide in the world, provides mechanical strength to the protective layers of nuts due to its crystalline structure [19,20]. Hemicellulose, on the other hand, acts as a supportive matrix with its branched monosaccharides [21], contributing to flexibility, and lastly, lignin is a complex amorphous polymer which is rigid, hydrophobic, and resistant [22].

Lignocellulosic components have a wide range of applications: cellulose and its derivatives are widely used in paper manufacturing, as well as in biomedical applications. Recently, there has been a growing interest in the production of nanocellulose [23,24,25], which is formed by physicochemical treatment in cellulose samples and is presented in the form of nanocrystals, nanofibers, or bacterial nanocellulose [26]. Each possess unique properties such as biodegradability and enhanced thermal and mechanical stability [27]. Nanocellulose has been applied to fields such as bioactive compounds or food packaging [28,29]. Hemicellulose, valued for its biodegradability and biocompatibility, serves as a sustainable material for bioplastics, pharmaceuticals, and biofuels [30]. Meanwhile, lignin, with its antioxidative and antibacterial properties, finds applications in biomedical fields and as a natural adhesive in the pellet industry [31,32].

However, due to their natural recalcitrance [33], to successfully isolate them, exhaustive extraction methods are required to separate and utilise these components effectively [34]. Therefore, understanding the intrinsic polysaccharide composition of these materials is essential for selecting the most suitable extraction method and optimising the recovery process [35]. In particular, cellulose stands out due to its valuable properties and diverse applications [36,37], making its extraction the central focus of this review.

To achieve this, an effort in the scientific community has been made in the search for different extraction and fractionation techniques. These methods, which can be physical [38] and chemical [39,40,41,42], range from a wide diversity of chemical compounds, as well as instrumental techniques, with the objective of not only facilitating the selective separation of lignocellulosic components but also enhancing their functional properties, thereby broadening their potential applications in food packaging [43] and biodegradable composites [44], among others.

As can be seen, nut by-products represent a significant source of lignocellulosic biomass, with large quantities generated annually as a result of nut production worldwide. However, the pre-treatment of high-lignin-containing lignocellulosic materials, such as nut by-products, has not been fully studied, and there is still much to be discovered and understood in terms of optimising processing methods [45]. While lignocellulosic components in other biomass sources have been extensively explored, nut by-products have received relatively less attention, particularly regarding novel and sustainable pre-treatment strategies. The objective of this review is to address this knowledge gap by providing a comprehensive state-of-the-art analysis of both conventional and emerging techniques for processing nut by-products. This review aims to evaluate the efficiency and environmental impact of these methods by compiling and analysing the existing literature, with a focus on enhancing cellulose extraction and minimising lignin content. By doing so, it seeks to highlight the opportunities and challenges associated with the sustainable utilisation of these by-products, ultimately contributing to the development of more environmentally friendly and efficient processing strategies. The aim in this work is not to only provide a comprehensive overview of the current approaches but also serve as a call to explore new horizons in cellulose extraction.

## 2. Methodology

**Figure 1:**
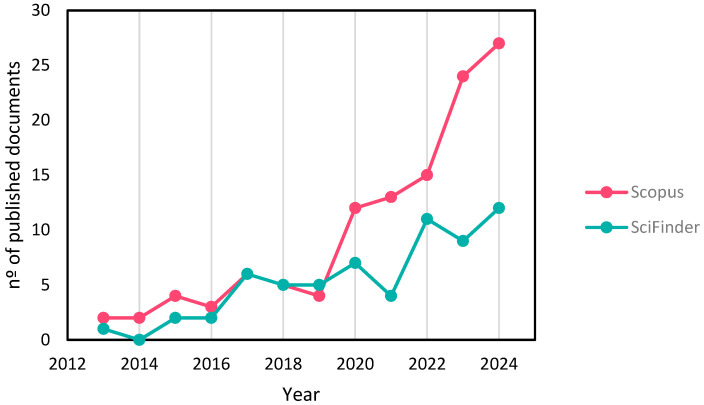
Published documents on cellulose extraction for valorisation of nuts, provided by Scopus and SciFinder.

Figure 1 presents the number of investigations published over the last decade for cellulose extraction from nut shells according to the Scopus and SciFinder databases (2013–2024), showing an increasing trend, mainly from 2019 onwards. This highlights the need for a comprehensive review to consolidate existing knowledge, compare different extraction methodologies, and identify research necessities. Despite the abundance of studies on lignocellulosic biomass valorisation, nut shells remain an underexplored source of cellulose, with significant potential for sustainable material applications.

An extensive literature review has been carried out using Google Scholar, Web of Science and Scopus databases in order to find all published papers (research papers and reviews) in English that involve all methods studied for fractionation of biomass tree and ground nut by-products, focusing specifically on cellulose isolation from this kind of lignocellulosic biomass. The searching strategy includes the use of “nut(s)”, “tree nut(s)”, “ground nut(s)”, “almond(s)”, “cashew(s)”, “walnut(s)”, “pistachio(s)”, “hazelnut(s)”, “Brazil nut(s)”, “chestnut(s)”, “Areca nut(s)”, “pecan(s)”, “macadamia(s)”, “shell(s)”, “husk(s)”, “cellulose isolation”, “cellulose fractionation”, “cellulose extraction”, “nut (re)valorisation” and “nut by-product(s)” as keywords. The term “nut” is not used in a botanical sense but rather as nut is commercially known, including both tree nuts and ground nuts. For a more detailed classification and definition, refer to Section 3 of this review.

It is worth noting that for some nut by-products, no studies on cellulose extraction have been conducted. This may be due to several factors, including a low shell yield that limits large-scale extraction or the presence of other compounds in the biomass with higher commercial value. For example, certain nut shells, such as those from pecans and Brazil nuts, are particularly rich in bioactive phenolic compounds, which have drawn greater research attention for their antioxidant and antimicrobial properties [1,46,47,48,49]. Others, such as cashews, are rarely studied for cellulose extraction due to their high content of cashew nut shell liquid (CNSL), a valuable phenolic oil used in bio-based resins and coatings [50,51,52].

In order to establish meaningful comparisons between different pre-treatment methods, results expressed as polysaccharide recovery and/or removal were compared (Equations (1) and (2)). Polysaccharide recovery, normally calculated for cellulose but in some cases for hemicellulose, reflects the percentage retained in the solid fraction after pre-treatment. This value can also represent the amount of recovered polysaccharide after its precipitation, which has previously been solubilised in the pre-treatment by an antisolvent method. On the other hand, polysaccharide removal provides information about the solubilisation of the compound in the reagent medium. These determinations are accurately calculated by considering the solid yield (Equation (3)) of the process.

However, in some studies where solid yield values were not provided, it was not possible to calculate these percentages precisely. In such cases, initial and final biomass composition values were used to compare the changes in cellulose, lignin, and hemicellulose content after the process applied (for each nut, “C” refers to the initial cellulose content, “H” to the initial hemicellulose content, and “L” to the initial lignin content, all expressed in g/100 g dm).

(1) $$Polysaccharide\;recovery\;\left(\%\;dm\right)=\left(\frac{Polysaccharide\;content\;after\;process\;(g)}{Polysaccharide\;content\;before\;process\;(g)}\times solid\;yield\;(\%\;dm)\right)\times 100$$

(2) $$Polysaccharide\;removal\;\left(\%\;dm\right)=100-Polysaccharide\;recovery\;\left(\%\;dm\right)$$

(3) $$Solid\;yield\;\left(\%\;dm\right)=\frac{solid\;content\;after\;process\;(g)}{solid\;content\;before\;process\;(g)}\times 100\;$$

## 3. Definition of Nut

The nut family conforms to a wide range of varieties, which can be classified into two main groups [14]: tree nuts—including almonds (*Prunus dulcis*), walnuts (*Juglans regia*), cashews (*Anacardium occidentale*), hazelnuts (*Corylus avellana*), pistachios (*Pistacia vera*), macadamia (*Macadamia integrifolia*), Brazil nuts (*Bertholletia excelsa*), chestnuts (*Castanea*), Areca nuts (*Areca catechu*), and pecans (*Carya illinoinensis*)—and peanuts (*Arachis hypogaea*), also called ground nuts, which grow in the form of legumes [53]. Peanuts, although not considered nuts, share a similar chemical composition and nutrient profile with them [54].

The term “nut” is botanically defined as a hard, indehiscent (said of fruits that do not split open when mature, but retain the seed till they decay), usually one-seeded fruit, often surrounded by a cupule [55]. However, not every “nut” that we commonly refer to as such fits this definition. Many of them are not “true nuts” botanically speaking (in fact, only hazelnuts and chestnuts are considered true nuts) but instead belong to different categories of fruits [56]. For example, almonds, walnuts, macadamias, and pecans are seeds enclosed in a hard inner layer (endocarp) of drupes (also called “dry drupes”), a one-seeded simple fruit developed from a superior ovary in which the innermost portion of the wall (endocarp) becomes hard and stony, the outermost part (exocarp) becomes a relatively thin skin, and the middle portion between the skin and the stone (mesocarp) becomes either fleshy or fibrous [57]. In the case of dry drupes such as almonds, the seed is the edible part, in contrast to fresh fruits such as apricot, where we eat the fleshy part. Also, it is interesting to note that cashew presents a distinct botanical structure despite being in the nut family, as the cashew is the seed that develops at the bottom of the cashew apple, which is also edible [58]. However, inside the double shell that protects the interior, there is a resin known as cashew nut shell liquid (CNSL), which can cause burning when in contact with human skin. This liquid is normally used in the industry in varnishes or lubricants [59]. Despite these botanical distinctions, the term “nut” is commonly used in a broader culinary and commercial sense to refer to the diverse group of both edible nuts and drupes [60].

In addition to these classifications, peanuts are often referred to as nuts, despite being botanically classified as legumes. They belong to the *Fabaceae* family, similar to lentils, beans, and peas. Peanuts grow in pods underground and are not tree nuts, yet they are nutritionally similar and used in cooking, like tree nuts [61,62].

## 4. Nut Production

**Figure 2:**
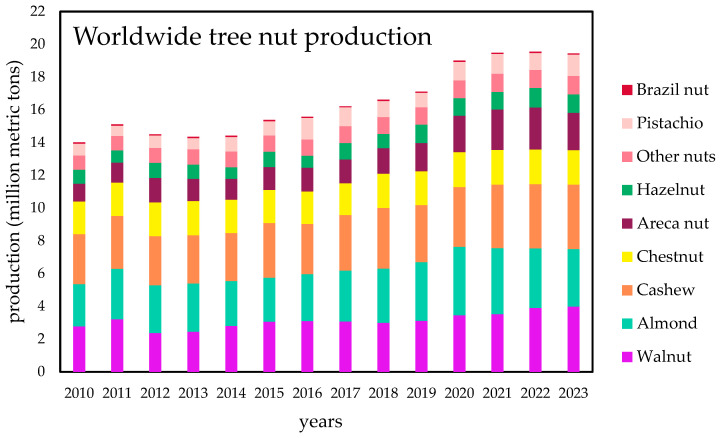
Global tree nut production over the years (2010–2023) [63]. Values are based on official and estimated values reported by FAOSTAT. “Other nuts” is a category used by FAOSTAT that includes minor tree nut species with relatively low individual production volumes, grouped for reporting purposes.

Global nut production has experienced a remarkable increase in recent years, rising from 12.92 million metric tons (MT) in 2014 to 17.16 million MT in 2023 [63] for tree nut products. Similarly, peanut production has also increased during the same period, from 43.78 million MT to 54.27 million MT. In Figure 2, a detailed graph illustrates the increase in production for each tree nut since 2010, excluding coconuts, whose production in millions of MT is much larger compared to the others, with an average of 62.22 million MT over the last ten years. Despite some fluctuations, a general increasing trend can be seen, all of them exhibiting a steady increase over time. The most extensively cultivated tree nuts are walnuts, almonds, and cashews [63], as reported also by Wojdyło et al. [64]. In contrast, Brazil nuts have maintained a lower production, with no significant increase, partly due to their limited global consumption, as they contain high levels of selenium [65]. Others, such as pistachios, can only grow in limited regions with specific climates [66].

**Figure 3:**
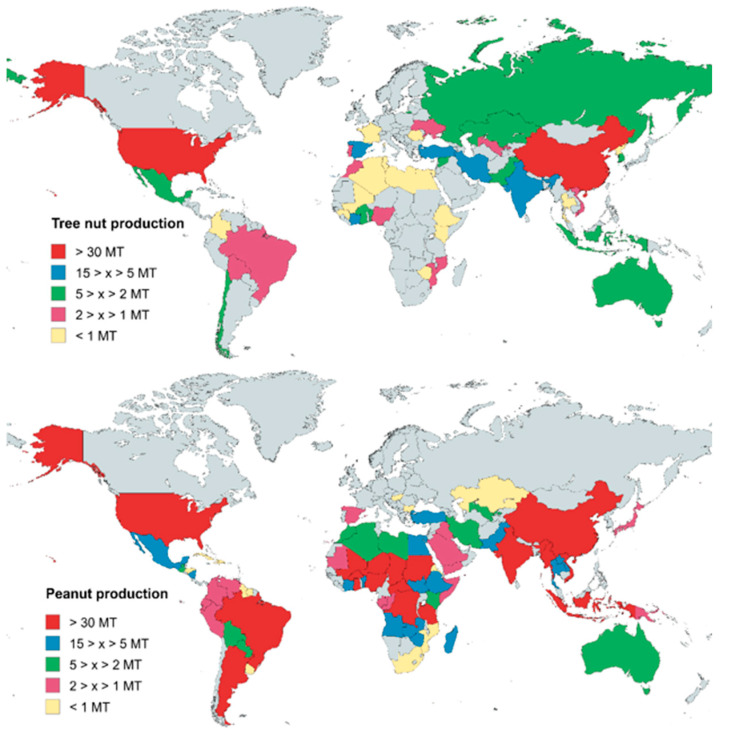
Geographical distribution of tree nut and peanut productions in 2023, by country. The values are based on official and estimated FAOSTAT (2025) data, and the countries are color-coded, highlighting the major producers in darker shades.

**Figure 4:**
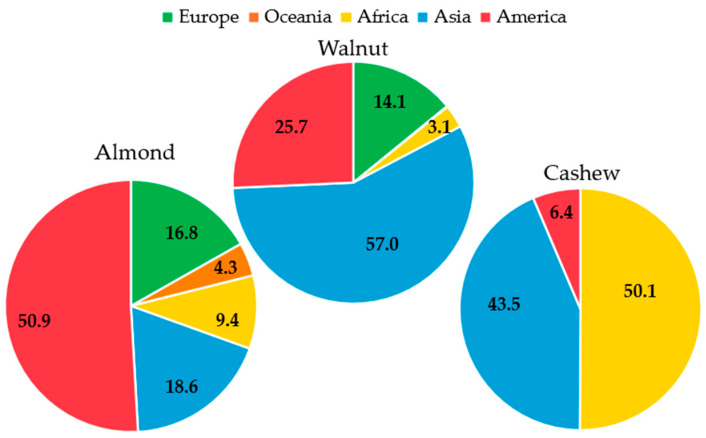
Geographical distribution of almond, walnut, and cashew nut crops, each of them representing different climate conditions (almond: temperate dry and hot summer and temperate dry and warm summer; walnut: temperate hot summer with no dry season and tropical rainforest and dry hot arid desert; cashew: tropical savanna and tropical monsoon) according to Koppen–Geiger classification).

A visual nut geographical distribution can be seen in Figure 3, where it can be appreciated that peanuts need more humid climates with warm temperatures, but tree nuts are more concentrated in mild, dry regions. A key factor behind their widespread production is their adaptability to diverse climatic conditions, particularly temperature and moisture availability, allowing them to be grown in multiple regions worldwide [67]. It is important to note that most nut crops undergo a dormancy stage in winter, a crucial adaptation to avoid damage [68]. According to the Köppen–Geiger classification, nut-producing regions can be categorised by different climate conditions [69]: when the dormancy mechanism is particularly essential, temperate species grow in places with hot and dry summers and mild and often wet winters (USA, Turkey, Spain). In this climate, the ideal nut crops are almonds, pistachios, and hazelnuts, where dry harvesting is a need [70] (temperate dry and hot summer and temperate dry and warm summer, in the Köppen–Geiger classification) [63]. However, where there is not a dry season and rainfalls occur (China, Brazil, Iran), this climate is suitable for nuts that require more moisture, such as walnuts, peanuts, or pecans (temperate hot summer with no dry season and tropical rainforest and dry hot arid desert, in the Köppen–Geiger classification) [63]. Other nuts, such as cashews or macadamias, are grown in warm-temperature places (tropical savanna and tropical monsoon, in the Köppen–Geiger classification) like India or Ivory Coast [63]. Figure 4 shows the geographical distribution of almond, walnut, and cashew nut crops, each of them representing one of these three different groups of climate conditions.

As has been said, many countries contribute to nut production, with China and India as the largest producers of ground nuts, representing 35.4 and 18.9% of the global value (54.3 MT) [63]. Additionally, global tree nut production (17.2 MT) is led by the United States, representing 20.1%, while China also stands in this category (18.5%) [63]. Other significant producers in both groups include Turkey, Iran, Ivory Coast, and Spain, which play a crucial role in both producing and exporting large quantities to meet the demand for direct consumption, food processing, and industrial applications all over the globe. These values are accompanied by a large quantity of harvested areas around the world. Particularly, in Spain, approximately 850.000 ha of agricultural land was dedicated to almond, walnut, and hazelnut cultivation in 2024 [71].

## 5. Nut By-Products and Their Importance

**Figure 5:**
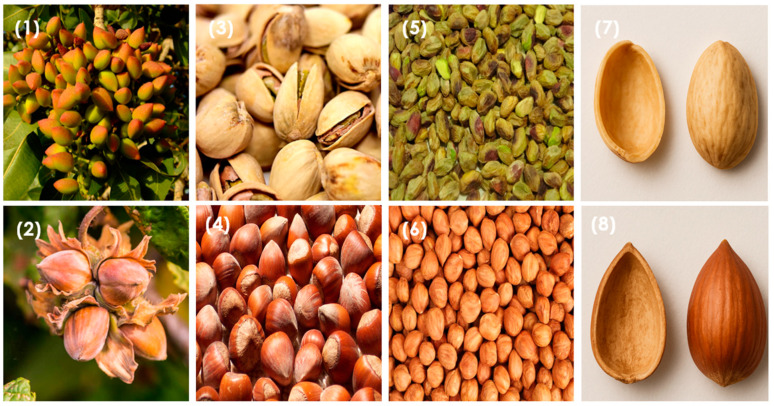
Different parts of pistachio and hazelnut crops. Fruits before harvesting, in the tree (**1**,**2**) [76,77], after harvesting, conserving its protective layer (**3**,**4**) [78,79], after hull or shell removing, revealing the kernel (**5**,**6**) [80,81], and its distinctive shell (**7**,**8**) [82].

**Figure 6:**
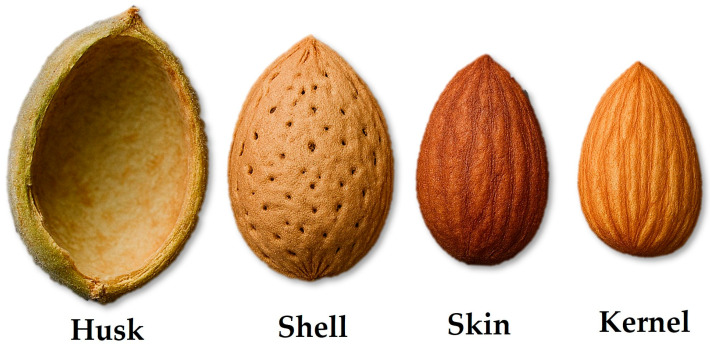
Different parts of an almond nut [82].

The nut industry generates not only the edible kernel used for eating or cooking but also various structural components that, although often considered as waste, form part of the whole fruit [72]. Two types of nuts (pistachios and hazelnuts) are presented in Figure 5, showing the different parts that constitute them before and after harvesting. Leaves, pruning fibers, and most of all, the protective layers of the kernel, are the main nut by-products, which are released and accumulated once harvested. These last are natural protective layers and are referred to as husk (epicarp and mesocarp), shell (endocarp), or skin (also referred to as *Testa* or seed coat) [73]: the husk protects the overall structure from the outside, normally with a significant hardness and thickness [74], in contrast to other fruits, where the mesocarp is fleshy and edible [73]. The shell, on the other hand, is a rigid and hard structure that envelops the kernel itself, and during development, it becomes also considerably hardened due to the presence of structural polysaccharides [75]. Finally, the skin directly surrounds the kernel and provides additional protection while also containing phytochemical compounds that contribute to the flavour and antioxidant properties of the nut [1,48]. As an example, the different parts of an almond can be appreciated in Figure 6.

The composition of these layers, which is well documented by numerous investigations in the field [83,84], can significantly change depending on the type of nut, being rich in different compounds such as vitamins, minerals, bioactive chemicals, fibre, lignin, proteins, polysaccharides, and even oils [85]. In order to introduce the kernel nuts into the industrial food chain, most of these by-products need to be removed [86].

Therefore, as the nut production increases, so does the generation of these by-products. Detailed percentages of husk, skin, shell, and kernel, relative to the total weight of the fruit, are shown in Table 1, where data, expressed on a dry-weight basis, were collected from different experimental measurements reported in the literature (see references on the table). It should be noted that the large deviations are probably due to differences among cultivars, as well as variations in growing conditions across regions [87]. Some nuts, such as hazelnuts, do not have a distinct husk, as their outer layer is already the shell [88]; in others, the husk percentage is included in the shell percentage.

Regarding the shell-to-kernel percentage, which varies widely among different nut species, chestnuts exhibit the lowest shell proportion due to their thin and lightweight shell [89], possessing a spiny and barbed outer layer [90]. In contrast, cashew nuts have the highest shell proportion, with a value of approximately 64% of the total weight [91]. All values are on a dry-weight basis.

**Table 1:** Husk, shell, skin, and kernel percentages of different types of nuts.

	Husk (%)	Shell (%)	Skin (%) [87]	Kernel (%)	Ref.
Almond	52.7 ± 8.05	32.76 ± 6.81	4–6%	14.58 ± 2.48	[92]
Areca Nut	Included in shell %	42.30 ± 2.76	Not reported	58.34 ± 2.65	[93]
Brazil Nut	-	49.82 ± 2.58	Not reported	50.18 ± 2.58	[94]
Cashew	Included in shell %	63.84 ± 6.46	5.36 ± 5.33	31.67 ± 7.14	[91]
Chestnut	Included in shell %	13.95 ± 1.92	Not reported	86.00 ± 1.92	[89]
Coconut	54.38 ± 0.7	15.18 ± 2.40	Not reported	-	[95]
Hazelnut	-	57.66 ± 7.77	2–3%	42.34 ± 7.77	[96]
Macadamia	Included in shell %	57.32 ± 1.18	Not reported	42.98 ± 0.79	[97]
Peanut	Included in shell %	31.03 ± 0.11	2.5–3%	68.90 ± 0.11	[98]
Pecan	Included in shell %	47.08 ± 2.95	2–5%	52.92 ± 2.95	[99]
Pistachio	Included in shell %	45.18 ± 7.02	9–11%	54.82 ± 0.65	[100]
Walnut	Included in shell %	50.55 ± 0.85	Not reported	49.45 ± 7.02	[101]

Although agricultural efforts have been historically made to select varieties with bigger kernel sizes for commercial purposes [102], the presence of a husk and/or shell remains essential for protecting and successful growing of the nut [15,16]. Consequently, as long as nuts are cultivated and consumed, the generation of by-products will be an unavoidable outcome [87]. However, to extend their shelf life, in some cases, the skin is intentionally preserved because it is safe for human consumption and possesses beneficial phytochemicals and antioxidant activity [49]. Xiao Peng et al. [103] demonstrated that nut skin was not genotoxic. Therefore, certain studies do not report an exact value respecting the total weight of the nuts, but a range. Nevertheless, the proportion of skin with respect to the total nut is relatively low, only reaching 11% for pistachios.

It is essential to understand the number of by-products generated annually by agro-industrial crops, referred to as agro-industrial by-products (AIBPs) [104]. Particularly, the disposal of nut by-products remains a significant challenge due to limited dumping spaces and environmental concerns associated with them [105]. Although various applications have been explored, they are not sufficient to fully integrate these residues into high-value chains. As a result, a considerable fraction continues to be discarded, highlighting the persistent issue of waste generation despite ongoing valorisation efforts.

In addition to the residues generated during the processing of shelled nuts, further waste is produced when they are marketed with the shell or husk. Although nuts have an extended life, strict standards about the quality of the whole nut are required to be launched on the market [106]. These criteria, known as quality factors, include the absence of foreign materials and physical defects in the husk or shells and appropriate dryness or colour uniformity, among other factors that could compromise the nut’s suitability for consumption [107]. As a result, a large quantity of nuts is labelled as unsuitable for the market, leading to the disposal of both the kernel and its non-edible fractions and contributing to the total by-products generated each year.

### 5.1. Nut By-Product Composition

**Figure 7:**
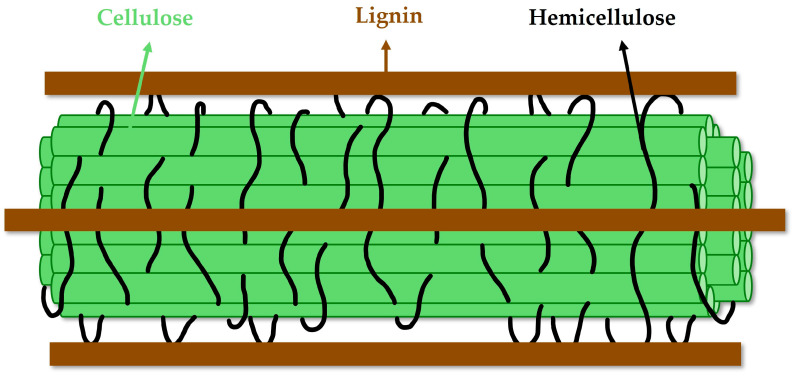
Graphical representation of lignocellulosic biomass structure, showing its three main components: cellulose, hemicellulose, and lignin.

Protective layers of nuts are composed of mainly cellulose, hemicellulose, and lignin, and they can also be referred to as lignocellulosic biomass, which is considered the most promising renewable resource on earth [108]. Lignocellulosic biomass can be defined as a three-dimensional combination of structural polysaccharides, present in the cell walls of plants (Figure 7) [109]. Its interest lies in extracting, isolating, or fractionating its precursors for highly valuable applications [110].

**Figure 8:**
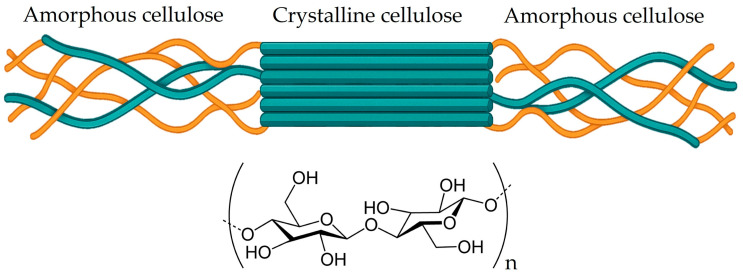
Visual representation of crystalline and amorphous domains of cellulose chains, and their chemical structure.

Cellulose is a linear carbohydrate polymer considered as the most abundant polymer on the planet. It is widely recognised for its exceptional rigidity and structural stability [20], primarily due to the strong network of hydrogen bonding and van der Waals forces between its repeating glucose units, (C_6_H_10_O_5_)_n_ [19]. These glucose monomers are linked by β-1,4-glycosidic bonds, forming long, unbranched chains that assemble into microfibrils. Within these microfibrils, crystalline and amorphous regions coexist (Figure 8), creating a dual-phase structure that influences the mechanical and chemical properties of cellulose [111,112]. The highly ordered crystalline domains contribute to cellulose’s strength and resistance to degradation, while the amorphous regions provide flexibility and increase accessibility for chemical modifications [113]. These microfibrils are intertwined in a hemicellulose and lignin matrix, creating larger macrofibrils that conform nut shells and husks [114].

In contrast, hemicellulose is a heterogeneous branched polysaccharide, composed mainly of xylan or xylose monomers, depending on the plant [21]. Hemicellulose plays a crucial role in cross-linking cellulose microfibrils, forming a double-helical ribbon structure around them [115]. Hemicellulose interacts with lignin forming a dense network through non-covalent interactions and covalent linkages. Lignin is an amorphous, highly branched heteropolymer which derives in hydrophobicity and chemical complexity [116], creating a protective and strong layer around cellulose fibers.

Together, cellulose, hemicellulose, and lignin form an intricate and highly resistant matrix that determines the physical and chemical properties of lignocellulosic biomass [117]. Their strong interactions between these components, a phenomenon known as recalcitrance [33], have evolved as a natural defence mechanism in plants to fight external and environmental degradation [118]. Therefore, this compact structure raises significant challenges in the extraction and subsequent up-cycling of its components [119]. Because of that, intense investigation has been carried out to develop efficient fractionation and valorisation strategies.

Nut shells are heterogeneous lignocellulosic materials, and their chemical composition can vary significantly depending on the species and cultivation and maturation conditions. In particular, the lignin, hemicellulose, and cellulose contents differ widely among nut types, which directly affects their behaviour during the extraction processes. For instance, lignin concentration is not only species-dependent but also influenced by environmental factors and the maturation stage of both the plant and the fruit [120,121]. Moreover, it is important to consider that the analytical methods used for the characterisation of the lignocellulosic components can vary between studies, which may lead to variations in the reported values.

Some nut shells, like peanut, pistachio, and walnut shells, tend to exhibit higher cellulose contents [99,101,102], making them more attractive for cellulose isolation. Conversely, almond and hazelnut shells typically present lower cellulose levels and higher lignin contents [92,96], a characteristic that contributes to their increased mechanical strength and rigidity. 

While the focus on this review is on organic molecules, it is important to note that nut shells are also rich in inorganic compounds, including potassium, calcium, and magnesium.

### 5.2. Applications of Nut By-Products

Nut by-products have traditionally been used in different applications, focusing on the exploitation of the material without any further modification except reducing its particle size due to handling issues (see Section 6 for a detailed explanation). One of the most common ways to use nut by-products, due to their high lignin and cellulose content, is as feedstocks for pyrolysis [122], producing char with a high carbon content that can serve as a renewable energy source. Demirbaş [123] studied the kinetic parameters of the pyrolysis of hazelnut shells of different particle sizes and also in the presence of a K_2_CO_3_ catalyst. However, the potential of nut by-products as fuels is limited in some cases due to the high potassium content in them, such as in almond shells. Potassium can interfere with their use as a solid biofuel, particularly at elevated temperatures, due to risks of furnace corrosion and ash-related problems such as slagging or clogging [124,125]. Consequently, certain nut shells are not considered suitable for combustion-based energy production, reinforcing the need to explore alternative valorisation pathways for these by-products.

Nut by-products are also used as natural fertilisers [126], as they can release nutrients as they decompose over time. Karagöktaş et al. [127] demonstrated that composted pistachio husks can be successfully incorporated into soils, decreasing the pH of the matrix and therefore increasing the phosphorous and zinc bioavailability. Additionally, nut by-products have been used as animal feed due to their fibre and nutrient content [128]. For instance, Swanson et al. [129] investigated the inclusion of almond husks in ruminant diets, improving their nutritive value as an alternative feed ingredient without compromising animal health or performance.

However, these uses typically involve simple mechanical processing and they do not fully exploit the intrinsic value of their chemical composition. Targeted extraction techniques enable the separation and purification of specific compounds, thereby maximising their functionality and industrial relevance. Therefore, it is possible to isolate and refine these interesting compounds such as cellulose for specialised applications, enhancing their functional properties and expanding their industrial value. This transition from bulk utilisation to component-specific valorisation not only gives an interesting point of view, improving resource efficiency but also aligns with the circular economy model, promoting the transformation of agricultural residues into high-value products.

## 6. Sample Preparation

The isolation of cellulose from lignocellulosic biomass requires preparatory steps to optimise extraction efficiency. Particularly, as nuts develop in open environments, they can accumulate impurities and pollutants such as dust, microbial residues, and chemical compounds, which can interfere with sample processing. Therefore, an initial washing step (distilled water, mild surfactants or diluted acid or alkaline solutions) is necessary to remove these impurities and avoid microorganism growth and therefore ensure stable storage [130] and further processing.

Following washing, mechanical size reduction is essential to improve accessibility to delignification agents and also for sample homogenisation [131]. Nut shells, like other lignocellulosic materials, present a highly compact and rigid structure, where larger particles may hinder the efficiency of subsequent treatments [132]. Reducing particle size increases the surface area-to-volume ratio, facilitating the diffusion of chemical reagents and enhancing cellulose separation from lignin and hemicellulose [133,134]. Therefore, particle size reduction arises as a mandatory step before any lignocellulosic biomass treatment [135]. Various mechanical techniques can be used, including crushing or milling (e.g., ball milling, hammer milling, or disc milling). Sieving is often applied to ensure a uniform particle size distribution, minimising variability in process efficiency.

The impact of particle size on cellulose extraction has been widely investigated [136], with studies generally indicating that smaller particles lead to improved cellulose recovery due to enhanced chemical penetration [137,138].

In some cases, researchers consider it necessary to remove extractive compounds (free sugars, fatty acids, waxes, and phenolic compounds) before compositional analysis, as these compounds can interfere with both the accurate quantification of lignocellulosic composition [46,139,140,141]. These are commonly removed through Soxhlet extraction using water, ethanol, n-hexane, or petroleum ether, among others, for a duration of 4 to 8 h, ensuring a more reliable characterisation and processing of the biomass.

## 7. Extraction Methods

The efficient isolation of structural polysaccharides of lignocellulosic biomass, particularly cellulose, has emerged as a significant research focus in recent years [127]. However, several challenges must be addressed before achieving its effective extraction. One of the major obstacles is the high lignin content in the material, which surrounds cellulose macrofibrils and acts as a barrier [142]. Lignin is considered the most difficult component to remove in lignocellulosic biomass [143]. Once this amorphous component is eliminated from the matrix, the surface area increases, as well as its crystallinity [144]. Consequently, extensive efforts have been carried out to develop delignification strategies that are both efficient and environmentally sustainable. A deep understanding of these techniques is essential to identify the most promising approaches for achieving sustainable cellulose isolation.

Lignocellulosic biomass processing involves different treatments which are normally accompanied by one or various pre-treatment steps. This preparation step is considered the most expensive one in lignocellulosic fractionation [145]; therefore, an ideal strategy should not only enhance cellulose accessibility but also minimise the formation of inhibitory compounds and costs of the process, equipment [146], and energy demand [147,148].

Conventional physicomechanical processes which reduce the particle size and homogenize the sample (e.g., ball milling, grinding) are already applied when nut by-products are used in traditional applications as a whole [38], as mentioned in Section 6. However, when the objective is to take advantage of its main components, particle size reduction is seen as a preparation step. In conjunction with further physicochemical pre-treatments, which modify the biomass structure and improve cellulose accessibility [149], recalcitrance is successfully mitigated [150].

Other physical methods, such as microwaves [151] and ultrasonication [141], are often employed. However, the main disadvantage is the high energy input needed. Chemical methods, including acid or alkaline processes [152], effectively remove lignin but may generate inhibitory by-products. A combination of these two methods, known as physicochemical approaches (e.g., autohydrolysis [153]), offers a balance by enhancing lignin weakening or removal while reducing harsh chemicals. Also, the deep eutectic solvents (DESs) [154] approach has gained attention in the last decades. A balance must be achieved between pre-treatment severity and cellulose preservation, as overly aggressive conditions can degrade cellulose [155], while milder treatments may leave residual lignin, limiting accessibility. Optimising these processes is essential to maximising cellulose yield while minimising energy consumption and environmental impact.

Following the pre-treatment stage, bleaching processes are typically applied to achieve further delignification and enhance the purity of the isolated cellulose. These steps often involve oxidative or alkaline treatments—such as sodium chlorite under acidic conditions [156] or hydrogen peroxide in alkaline media—that target residual lignin structures. The oxidative cleavage of aromatic rings and the disruption of ether and carbon–carbon linkages within the lignin polymer facilitate its solubilisation and removal [157,158].

It is also important to note that alongside cellulose isolation, one of the strategies to obtain a highly pure material is the removal of the surrounding hemicellulose in the cellulose matrix [159]. Hemicellulose extraction and subsequent depolymerisation can be achieved through a variety of thermochemical and enzymatic strategies, each influencing the structural integrity and distribution of the resulting products. Mild hydrothermal pre-treatment, such as liquid hot water pre-treatment, typically cleaves the labile glycosidic linkages and deacetylates the side chains, leading to the formation of soluble oligosaccharides [160]. These compounds, such as xylose or mannose, are widely studied for their prebiotic properties, promoting the growth of beneficial gut microbiota [160,161]. More severe conditions, including dilute acid hydrolysis, can further break these oligomers into monosaccharides, predominantly xylose, along with degradation products like furfural and formic acid [162,163], which can be used in the biofuel industry or for synthesising resins and bio-based polymers [164]. Moreover, organosolv processes use organic solvents under acidic conditions to solubilise hemicellulose, yielding xylose-rich fractions and acetylated oligosaccharides [165].

Although numerous extraction techniques are currently under investigation for the recovery of all main lignocellulosic fractions—including hemicellulose and lignin—this review focuses primarily on cellulose. Given these considerations, the following sections will explore a range of conventional and emerging strategies for cellulose isolation from lignocellulosic nut by-products. Each method will be examined with regard to its fundamental principles, processing conditions, and performance in terms of lignin removal and cellulose recovery.

Table 2 constitutes the structural backbone of this review, offering a comprehensive synthesis of the selected studies. It enables direct comparison of key parameters and outcomes across different approaches, serving as an essential reference point throughout the discussion. Particular emphasis will be placed on the comparative analysis of the techniques, with a focus on their respective benefits, drawbacks, and environmental implications.

### 7.1. Alkaline Pre-Treatment

Alkaline pre-treatment is one of the most conventionally used methods for lignin removal and therefore for cellulose purification, both historically and in modern industrial applications [144].

Alkaline treatments, typically carried out with NaOH, KOH, or NH_3_, selectively remove non-crystalline compounds through saponification reactions [180]. In the process, the reagents swell the cell walls of the lignocellulosic sample, breaking down the ester linkage between hemicellulose and lignin and increasing porosity and cellulose crystallinity, enhancing glucose yield [181]. As the concentration of alkaline reagent increases, lignin removal efficiency improves due to the more aggressive chemical environment, which reduces the recalcitrance of the lignin structure and facilitates its depolymerisation.

Within the different investigations carried out, alkaline treatments performed with NaOH were the most common for nut shell processing. Zheng et al. [179] treated walnut shells (initial composition of C: 27.4 g/100 g dm; H: 31.3 g/100 g dm; L: 36.3 g/100 g dm) and obtained a cellulose content of 56.6 g/100 g dm after four cycles of 2% NaOH solution for 4 h at 100 °C. This purification was achieved thanks to the selective hemicellulose removal effect of NaOH. However, residual lignin was still on the matrix; therefore, a subsequent 1.7% NaClO_2_ bleaching for 6 h at 80 °C was applied, successfully removing it completely and obtaining a cellulose content of 87.9 g/100 g dm.

The importance of the intensity of this treatment can be assessed by comparing it with the study carried out by Kasiri et al. [174], where pistachio shells (initial composition of C: 38.1 g/100 g dm; H: 31.4 g/100 g dm; L: 23.6 g/100 g dm) underwent the same NaOH pre-treatment but only for a single cycle. Despite applying the same bleaching process, the milder pre-treatment resulted in lower efficiency, yielding a final cellulose content of 71.3 g/100 g dm and a lignin content of 8.2 g/100 g dm. In comparison, treatment of moderate intensity for hazelnut shells (initial composition of C: 32.1 g/100 g dm; H: 17.9 g/100 g dm; L: 38.7 g/100 g dm)—3% (*v*/*v*) NaOH for 3 h at 80 °C, followed by three 2.7% NaClO_2_ bleaching cycles at 80 °C for 1 h each—produced intermediate purification results, with a final cellulose content of 70.8 g/100 g dm [170]. This comparison highlights the critical role of selecting an appropriate pre-treatment strategy to achieve the desired cellulose purity. A similar trend can be seen in Hoşgün et al. [168], where a 2.25% NaOH solution for 30 min and 60 °C treatment was carried out for hazelnut shell cellulose isolation (initial composition of C: 16.7 g/100 g dm; H: 16.7 g/100 g dm; L: 51.3 g/100 g dm). Due to milder conditions, the final cellulose content was 18.1 g/100 g, only 2% higher than the initial value, requiring further bleaching.

Bleaching steps were also needed in Chen et al.’s research [140] to extract cellulose from peanut shells (initial composition of C: 45.3 g/100 g dm; H: 8.8 g/100 g dm; L: 30.3 g/100 g dm). First, an 8% H_2_O_2_ solution at 50 °C was applied to the raw matter as an alkaline pre-treatment for 8 h, followed by two bleachings: one with a 2% NaClO_2_ solution (4 h, 75 °C) and another with a 2% NaOH solution (24 h, r.t.). The sequential process obtained a cellulose value of 79.7 g/100 g and the removal of almost all lignin and hemicellulose from the matrix. These results agree with other previously reported results of alkaline pre-treatment followed by bleaching [170,174,179].

Comparing alkaline pre-treatments with other techniques showed that different methods can selectively remove one polysaccharide or another. In Hoşgün et al. and Hoşgün and Bozan’s investigations [168,169], alkaline pre-treatment effectivity was compared with acid and hydrothermal pre-treatment, for hazelnut shells (initial composition of C: 16.7 g/100 g dm; H: 13.3 g/100 g dm; L: 51.3 g/100 g dm) at different times and temperatures. Alkaline treatment carried out with a 2.25% NaOH solution for 0.5–1 h at 60–150 °C effectively solubilised lignin by cleaving its ester bonds [182] (49.7 and 79.6% of lignin removal for pre-treatment at 60 and 150 °C, respectively) but partially retained hemicellulose (27.0 and 52.2% dm of removal for pre-treatment at 60 and 150 °C, respectively). In contrast, dilute acid pre-treatment with 0.5–1% H_2_SO_4_ for 15–30 min at 120 °C selectively solubilised hemicellulose (88.5% dm of removal on average), with the removal effect enhanced by the increased efficiency of mass and heat transfer under high-temperature conditions [183]. Autohydrolysis, using water at 120 °C for 15 min, was also selective for hemicellulose rather than lignin, as in dilute acid pre-treatment. Consequently, alkaline pre-treatment better delignified the sample and resulted in a greater cellulose yield, obtaining a value of 37.33 g/100 g. This can be attributed to the retention of hemicellulose, particularly glucomannans, which preserve the cellulose fraction [184]. These results align with previous studies highlighting the direct relationship between temperature, process time, and treatment previously mentioned [168,170,174,179].

Another wise option is to combine alkaline pre-treatment with other physicochemical processes in sequence, such as autohydrolysis and organosolv treatments, yielding better results in cellulose purification [185], as well as avoiding the generation of by-products such as furfural or acetic that can inhibit further cellulose applications [186].

A recent study on the pre-treatment of hazelnut shells was carried out, where researchers explored how different combinations can impact the chemical composition of biomass [168]. Among single pre-treatments (alkaline (AP), dilute acid (DAP), and hydrothermal (LHW), AP achieved the highest lignin removal of 49.6%, significantly improving biomass accessibility for enzymatic hydrolysis [187,188]. However, it resulted in the lowest cellulose recovery (85.4% dm) compared to DAP (96.7% dm) and LHW (98.0%), due to its ability to solubilise amorphous regions in cellulose chains [189]. When combined with LHW, an interesting effect occurred depending on the order of application: when AP was applied first, substantial lignin removal (60.7% dm) was achieved due to the synergistic β-O-4 bond cleavage by AP and the hot, pressurised water action of LHW, yielding 79.1% dm cellulose recovery. However, reversing the sequence reduced lignin removal to 46.7% dm, likely due to the recondensation of lignin droplets under high temperature and pressure, which weakened the AP’s delignification effect. Additionally, since AP is effective in removing lignin, combining it with a more selective method for hemicellulose, like DAP, further improves biomass purification. Applying AP before DAP resulted in 55.5% dm lignin removal, as lignin acts as a barrier that, once eliminated, allows the dilute acid to penetrate and solubilise hemicellulose. Reversing the order, however, was less effective due to the lignin recalcitrance.

Despite its effectiveness, this approach has several limitations, particularly the generation of by-products such as various salts, which are difficult to remove from the reaction medium. These environmental and operational challenges have driven the search for more sustainable and eco-friendly alternatives to improve the efficiency and environmental impact of lignocellulosic biomass processing. Overall, while alkaline treatment remains a widely used and effective method for delignification, its limitations in selectivity and environmental impact highlight the need for alternative approaches that improve lignin removal while preserving cellulose integrity.

### 7.2. Hydrothermal Pre-Treatment

In the search for greener alternatives to the most common methods, hydrothermal pre-treatment, also known as subcritical water extraction (SWE) or autohydrolysis, relies solely on water as the unique extraction solvent, making it cost-effective and readily available [190]. The process involves heating water below the subcritical point (between 100–374 °C and 1–22 MPa) [191]. Under these conditions, water undergoes significant changes in its physicochemical properties, allowing it to act as an adjustable solvent [192]. As the temperature increases, the strong hydrogen bonding network in water weakens, leading to a reduction in its dielectric constant [193]. Consequently, its polarity decreases, making it similar to more apolar solvents such as ethanol. Simultaneously, its viscosity and surface tension decrease, enhancing diffusion through the biomass matrix and improving efficiency [194,195]. Pressure is normally maintained primarily to raise water’s boiling point, as the changes in pressure have minimal impact on extraction efficiency [196].

During the process, hemicellulose present in nut by-products undergoes selective hydrolysis at around 180 °C, while cellulose remains stable until higher temperatures (around 220 °C) [197]. As hemicellulose breaks down, it releases organic acids that dissolve in water, increasing the acidity of the solution and contributing to the autohydrolysis effect by further enhancing the hydrolysis process. Lignin, on the other hand, suffers its breakdown into smaller phenolic compounds that can be more easily removed in further treatments [198]. Therefore, as a sustainable and efficient extraction method, hydrothermal pre-treatment has demonstrated significant potential across various application fields [192].

Recent research has demonstrated the potential of hydrothermal pre-treatment for the fractionation of lignocellulosic components of nuts. In Gil-Guillén’s et al.’s study [40], SWE was applied at 160 °C and 180 °C to almond shells (initial composition of C: 26.8 g/100 g dm; H: 23.6 g/100 g dm; L: 21.2 g/100 g dm). Significant hemicellulose degradation became evident only at 180 °C (hemicellulose value of 11.6 g/100 g dm), corroborating the initial temperature for its degradation (removal rate of 21.4% dm and 55.8% dm after 160 °C and 180 °C for 30 min treatment, respectively). This aligns with the theoretical understanding of hydrothermal pre-treatment, where increased temperature promotes greater hemicellulose hydrolysis [197] due to the increase of protons and hydroxyl ions in water, accelerating the depolymerisation [199,200]. Consequently, cellulose purification reached 41.7 g/100 g dm in the best conditions. This trend was also observed in Hoşgün et al.’s work [168], where they aimed to extract cellulose from hazelnut shells (initial composition of C: 16.7 g/100 g dm; H: 13.3 g/100 g dm; L: 51.3 g/100 g dm), where a lower temperature of extraction gave a lower hemicellulose removal (51.5% dm after 30 min at 120 °C). Following this behaviour, Surek and Buyukkileci [153] found a higher removal hemicellulose rate, also from hazelnut shell (92.4% dm) when increasing temperature up to 190 °C, having a final cellulose value of 57.7 g/100 g dm (initial value of 34.7 g/100 g).

Despite these observed trends, the impact on lignin content varied between the studies. In Gil-Guillén et al.’s work [40], lignin content decreased, but not by much (average 23.8% dm of removal after hydrothermal pre-treatment for 160–180 °C for 30 min). Conversely, in the studies of Surek and Buyukkileci [153] and Hoşgün et al. [169], despite not having the same temperature, the content in lignin increased as hemicellulose was selectively removed (14.9% dm and 12.6% dm of lignin removal, respectively). Similar findings were presented in Gullón et al.’s work [167], where chestnut shells (initial composition of C: 20.6 g/100 g dm; H: 10.5 g/100 g dm; L: 44.6 g/100 g dm) samples were treated with water at 180 °C in a stainless-steel reactor, giving a 16.6% dm of lignin removal and a final cellulose value of 26.0 ± 1.6 g/100 g dm.

However, although the removals were significant, the overall conclusion indicated that additional processing steps were necessary to achieve a high cellulose purification, labelling hydrothermal as a pre-treatment method. Given these results, further bleachings were applied in Gil-Guillén et al.’s investigation [40], resulting in a cellulose purity of up to 83.7 g/100 g dm using a 1.7% NaClO_2_ bleaching for seven times.

It has been demonstrated that hydrothermal methods, compared with traditional ones, give higher yields although having simpler strategies [201]. Pre-treatments such as alkaline or acidic, with more aggressive chemicals [168,169] underperform compared to hydrothermal pre-treatment in terms of cellulose isolation, offering a more efficient alternative (detailed in Section 7.1 and 7.3). However, other emergent methods such as deep eutectic solvents (DESs), in comparison with hydrothermal pre-treatment, showed a higher purification efficiency of cellulose for walnut shells, giving a final cellulose value of 83.5 g/100 g dm. Similar differences were observed when ChCl:OA DES was applied to coconut husk in Debiparna et al.’s work [202] (detailed in Section 7.5). Despite this, it is important to note that the DES method requires a significantly longer processing time, whereas SWE is completed in only 30 min.

Although the application of SWE for cellulose isolation from nut by-products has been explored, its effectiveness has also been demonstrated in other areas of agro-industrial waste valorisation. For instance, phenolic compounds have been efficiently extracted with SWE from potato peels [203] and kiwi pomace [204].

### 7.3. Dilute Acid Pre-Treatment

Dilute acid pre-treatment is considered an interesting strategy for lignocellulosic biomass processing due to its high efficiency at an industrial scale and its economic attractiveness [205,206,207]. This method can be explained as an evolution of hydrothermal pre-treatment, as it improves the process by incorporating a low concentration of acid (H_2_SO_4_, HNO_3_, or HCl) into the aqueous solution, which accelerates the cleavage of glycosidic bonds of hemicelluloses without requiring autohydrolysis [208], facilitating the breakdown of hemicellulose into monosaccharides while also altering lignin structure [209]. However, although the effectiveness of this process has been proven, it can generate inhibitory compounds when exposed to an acid medium, normally derived from xylose such as furfural [210]. These inhibitory compounds must be carefully managed to optimise biomass processing and further applications.

The effectiveness of dilute acid pre-treatment has been demonstrated in several investigations, which not only optimised cellulose recovery but also demonstrated superior selectivity for hemicellulose removal. In Hoşgün and Bozan’s work [169], a 1% H_2_SO_4_ solution at 120 °C for 15 min for hazelnut shells was applied (initial composition of C: 16.7 g/100 g dm; H: 13.3 g/100 g dm; L: 51.3 g/100 g dm) and resulted in 68% dm of hemicellulose removal, enhancing cellulose content (92.2% dm of cellulose recovery). Similar findings were shown in Hoşgün et al.’s study [168], where a lower acid concentration (0.5% H_2_SO_4_) was applied for a longer time (30 min), yielding almost the same cellulose content (21.3 g/100 g dm). This highlights the importance of balancing key factors, such as acid concentration, temperature, and time, to optimise the process in a more sustainable and energy-efficient way. In comparison with other treatments such as alkaline and steam methods, different results were observed: 2.25% NaOH alkaline pre-treatment resulted in the best lignin removal, in the same conditions (73.3% dm), while dilute acid pre-treatment was more selective to hemicellulose removal, as shown in Section 7.1. One important aspect to note is that increasing temperature and residence time during pre-treatment may lead to cellulose degradation [211,212]. While optimal cellulose recovery was observed under milder conditions (120 °C, 15 min), harsher conditions, such as 200 °C for 60 min, reduced recovery due to cellulose loss in solubilisation in the acidic medium (30.6% dm of cellulose recovery). Also, as was seen with alkaline pre-treatment, dilute acid pre-treatment can be successfully combined with other methods [168,169] (detailed in Section 7.1).

### 7.4. Ionic Liquid (IL) Pre-Treatment

Ionic liquids (ILs) are a class of solvents used in the pre-treatment and fractionation of lignocellulosic biomass, due to their unique physicochemical properties. They possess a low vapour pressure accompanied by high thermal and chemical stability and have great tunability [213]. In particular, ILs have demonstrated a remarkable capacity to disrupt the extensive hydrogen bonding network within cellulose, converting the crystalline regions into amorphous structures and thus enhancing fractionation efficiency in nut by-products [214,215].

They are entirely composed of ions, and their properties can be tailored by modifying their cation–anion combinations. Common cations include imidazolium, pyridinium, ammonium, and phosphonium, while typical anions are Cl^−^, [BF_4_]^−^, [PF_6_]^−^, [AcO]^−^, and [TF_2_N]^−^ [216]. Imidazolium-based ILs are widely used due to their simple synthesis and high catalytic activity, though they may degrade under alkaline conditions. ILs are also classified as protic or aprotic, with protic ILs showing higher conductivity and reactivity [217]. Despite their potential, the corrosivity, toxicity, and cost of ILs limit their industrial application, resulting in a growing interest in alternatives such as deep eutectic solvents (see Section 7.5).

The fractionation process generally involves dissolving raw biomass directly in an ionic liquid, followed by selective recovery of cellulose, lignin, or other components using antisolvents such as water, ethanol, or acetone. In the study by Carneiro et al. [218], peanut shells (initial composition of C: 33–49% dm; H: 9–19% dm; L: 21–34% dm) and chestnut shells (initial composition of C: 20–32% dm; H: 15–22% dm; L: 16–31% dm) were treated with two imidazolium-based ILs: [1-Ethyl-3-methylimidazolium acetate], also called [Emim][CH_3_CO_2_] and [1-Butyl-3-methylimidazolium hexafluorophosphate chloride], also known as [Bmim][Cl]. After 22 h, the samples showed a higher dissolution efficiency in [Emim][CH_3_CO_2_] IL, with no significant differences observed between the two temperatures tested (90 and 125 °C). Chestnut shells were more easily solubilised than peanut shells, likely due to their higher content of bioactive compounds (phenols and tannins) that are more readily dissolved in ILs. Cellulose was subsequently recovered by precipitation using an acetone/water (1:1) antisolvent mixture. Interestingly, cellulose recovery was higher for peanut shells (95% dm) compared to chestnut shells (75% dm), which may reflect the higher initial cellulose content in peanut shells. Delignification was not quantified in the study, which prevented direct comparison with other methods. However, an interesting parameter was reported: the purity of the precipitated cellulose-rich fraction was relatively low for both cases (about 50%), likely due to co-precipitation of non-cellulosic components or traces of residual IL, even after extensive washing. This is one of the main drawbacks in ionic liquid pre-treatment for lignocellulosic biomass. Moreover, ILs are reported to be over 80% more expensive than other similar solvents, such as DESs [219].

In Teh et al.’s investigation [172], IL pre-treatment was applied in macadamia shell (cellulose and hemicellulose not reported, initial composition of L: 35.5 g/100 g dm) using [Emim][CH_3_CO_2_] for 18 h at 110 °C, obtaining a successful dissolution of the shell. After precipitation of the cellulose-rich fraction (68.5% dm of recovery) by the antisolvent technique (using water-acetone and methanol; both showed good precipitation) the lignin value partially decreased (29.2 g/100 g dm). Notably, although similar conditions to those used in Carneiro et al.’s report [218] were applied, the cellulose recovery was lower, likely due to the shorter treatment duration. This underscores the importance of thoroughly optimising all parameters in IL-based pre-treatment processes to maximise cellulose yield and improve delignification efficiency.

Ionic liquid pre-treatment was also employed by Debiparna et al. [202] for cellulose extraction from tender coconut husks, as coconut has no distinct shell (initial composition of C: 34.9 g/100 g dm; H: 27.2 g/100 g dm; L: 37.1 g/100 g dm), using [Emim][Cl] under milder conditions (4 h, 70 °C). This method, referred to in the study as ionic solvent delignification, achieved a high cellulose recovery of 71.6% dm, and its purity was high, at 95.8%. The higher purity suggests that high temperatures may support impurity formation such as residual IL co-precipitation, as happened in Carneiro et al.’s study [218]. The investigation also compared this approach with two alternatives: a greener deep eutectic solvent (choline chloride–oxalic acid, 1:1, 90 °C, 4 h), which yielded the highest cellulose recovery (87.5% dm), and a more conventional alkaline peroxide delignification (H_2_O_2_/NaOH, 80 °C, 3 h), which resulted in a lower recovery (71.6% dm). The purities were similar to the IL method (average of 91.5%).

It has to be noted that the mentioned studies about IL method for cellulose isolation fundamentally rely on dissolving the entire biomass matrix, followed by selective precipitation of the cellulose desired fraction. However, this comprehensive solubilisation can be considered a drawback, as it often leads to the co-precipitation of undesired components, compromising the purity of the recovered material—even when milder conditions are used to mask this effect. In contrast, a stepwise isolation strategy, where undesired fractions such as lignin or extractives are selectively solubilised and removed, offers a better controlled and efficient route for obtaining cellulose.

In this regard, protic ionic liquids, particularly those used in the IonoSolv process [220], offer an effective alternative by selectively dissolving lignin while preserving the cellulose structure in the sample. Protic ILs such as triethylammonium hydrogen sulphate have demonstrated efficient lignin solubilisation, enabling its recovery through antisolvent precipitation [221,222], where water is normally used [223]. This approach not only enhances cellulose purity but also opens up opportunities for lignin valorisation into high-value products like carbon fibres and adhesives. The ability to tailor pre-treatment severity and precipitation conditions further allows control over lignin molecular weight and polydispersity, highlighting the potential of lignin-targeting IL systems for more precise biomass fractionation.

### 7.5. Deep Eutectic Solvents (DESs) Pre-Treatment

Following the promising but often problematic use of ionic liquids for biomass dissolution, DESs, an emerging class of green solvents, are increasingly being recognised as an advantageous choice for pre-treatment methods in lignocellulosic fractionation, including cellulose extraction from nut shells. DESs, first introduced by Abbott et al. [224], consist of a combination of two or more substances with dual roles: one acting as a hydrogen bond donor (HBD) and the other as a hydrogen bond acceptor (HBA). A ternary DES can be also synthesised by adding an acidic hydrogen bond donor (AcHBD). These solvents are highly valued for their low toxicity, biodegradability, low cost, and excellent biocompatibility [225].

In the context of cellulose extraction from nut shells, DESs offer several benefits: first, they can break down the lignocellulosic matrix by efficiently cleaving ether bonds between phenylpropane lignin units [226]. Moreover, DESs have a lower melting point than the individual components due to self-associated intermolecular interactions, such as hydrogen bonding and Van der Waals forces [178]. Also, DESs are simple to prepare and require no additional solvents, except for water to adjust viscosity [227]. This simplicity, along with their reusability, reduces both economic and environmental costs, making them an appealing choice for sustainable processes.

Li et al. [154] carried out an investigation focussing on DESs, specifically, choline chloride (ChCl), a common HBA, in combination with four different HBDs, to assess the efficiency of pre-treatment with different reagents. The HBDs selected were oxalic acid (OA), lactic acid (LA), citric acid (CA), and p-toluenesulfonic acid (TsOH). Interestingly, the presence of chloride ions (Cl^−^) from ChCl played a critical role in enhancing xylose degradation, as halide ions are known to accelerate hemicellulose depolymerisation [228,229]. Ethylene glycol (EG) was also added, as its presence inhibits undesirable carbohydrate degradation [230]. The goal was to evaluate the impact of acidity, which is determined by the combined effect of the HBD and the HBA [231], on the efficiency in cellulose isolation from walnut shells (initial composition of C: 33.4 g/100 g dm; H: 22.9 g/100 g dm; L: 38.6 g/100 g dm). It was observed that, as the acidity of the DES increased (pKa TsOH > pKa OA > pKa LA > pKa CA), so did the percentage of cellulose obtained. However, this increase in acidity was accompanied by lower solid yields, indicating an overall decrease in cellulose recovery as the acidity of the HBD rises [232]. The best cellulose recovery, which was 90.8% dm, was obtained using ChCl–CA–EG (1:1:1) at 120 °C for 4 h as pre-treatment (final cellulose value of 60.5 g/100 g dm). A similar trend was observed in hemicellulose recovery, where higher acidity led to greater degradation of hemicellulose, giving results up to 45.8% dm for the same DESs (final hemicellulose value of 9.5 g/100 g dm). Despite the reduction in solid yield, a significant improvement in lignin removal was achieved as acidity increased. This is particularly important for cellulose purification, as lignin acts as a barrier to cellulose’s further applications. The best results in lignin removal (93.1% dm) were obtained with ChCl–TsOH–EG (1:1:1) at 110 °C for 2 h (final lignin value of 9.4 g/100 g dm). The results demonstrated that higher acidity in DESs facilitates greater delignification, despite having less cellulose purification, as was also seen by Sazali et al. [232] in oil palm biomass fractionation.

However, some findings highlight that pKa alone is not the only determinant of DES effectiveness. This is evident from the study done by Li et al. [180], where the same ChCl–TsOH–EG (1:1:1) DES was used at a lower temperature (90 °C) also in walnut shells (initial composition of C: 32.9 g/100 g dm; H: 22.5 g/100 g dm; L: 38.6 g/100 g dm), resulting in a lignin removal of 70% dm (20% lower than that achieved at 120°C). This demonstrates again the crucial role of temperature and also the influence of multiple process parameters in optimising the extraction performance for effective lignin removal and cellulose purification.

DESs were also a wise choice in Debiparna et al.’s study [202], where they successfully applied ChCl–OA (1:1) for 4 h at 90 °C (experiment referred to as DESD) in tender coconut husks (initial composition of C: 34.9 g/100 g; H: 27.2 g/100 g; L: 37.1 g/100 g) for the extraction of cellulose for the further synthetisation of nanoparticles. These authors compared its effectiveness with two other methods: ionic solvent delignification (IOD) ([Emim][Cl] as a reagent for 4 h at 70 °C), and an alkaline peroxide delignification (APD) using a 30% dm H_2_O_2_: 2.5 M NaOH solution (3 h, 80 °C). They found that DESD pre-treatment was the most effective of all three, yielding a cellulose recovery of 87.5% dm, similar to the one previously reported by Li et al. [154] (of 90.8% dm). The IOD method achieved a cellulose recovery of 71.6% dm, as was detailed in Section 7.4. The APD method only yielded 83.1% dm of cellulose recovery, making DESD the most efficient pre-treatment among the three.

An interesting strategy for peanut shell fractionation (initial composition of C: 36.4 g/100 g dm; H: 15.6 g/100 g dm; L: 25.0 g/100 g dm) [173] involved the utilisation of lignin-derived phenolic compounds as part of the DES system, enhancing sustainability and circularity. In this study, ChCl was mixed in a 1:1 ratio with a lignin-derived compound (guaiacol) for 3 h at 120 °C. The obtained results showed that DES alone did not significantly alter the composition. However, the addition of AlCl_3_·6H_2_O increased the concentration of Cl^−^ ions beyond those provided by ChCl, further enhancing biomass fractionation. As a result, lignin and hemicellulose removal improved, leading to higher cellulose purification yields, with removal rates reaching 93.2% for hemicellulose and 73.2% for lignin, respectively (ChCl:guaiacol:AlCl_3_·6H_2_O 1:1:0.07). However, cellulose recovery results (final value of ≈60% dm) were lower than the ones previously reported [41,202], probably because the higher temperatures induced a slight cellulose degradation.

### 7.6. Organic Solvent (Organosolv) Pre-Treatment

One of the most common alternatives to conventional methods for cellulose purification is the use of organic solvents [233]. These solvents, normally mixed with water, include alcohols (e.g., glycerol, methanol, ethanol), ketones (e.g., acetone, methyl isobutyl ketone), organic acids (e.g., acetic acid, oxalic acid), and aprotic solvents such as tetrahydrofuran. Sometimes, a catalyst such as H_2_SO_4_ or NaOH is added to accelerate its performance and reduce pre-treatment time [234]. The method involves the disruption of α- and β-aryl ether linkages [235], which result in the fragmentation and subsequent dissolution of lignin and also hemicellulose [236].

Ethanol, a low-boiling point alcohol, is widely used as a solvent in organosolv pre-treatment due to its cost-effectiveness, low toxicity, and high miscibility with water [237]. Studies have demonstrated that ethanol-based organosolv pre-treatment, especially when catalysed with sulfuric acid, effectively removes lignin and enhances enzymatic hydrolysis [238].

A comparative analysis of organosolv pre-treatment for nut shells has been carried out in different investigations, where notable differences can be appreciated. In the study done by Morales et al. [177], walnut shells (initial composition of C: 18.8 g/100 g; H: 27.7 g/100 g; L: 33.3 g/100 g) were subjected to organosolv treatment using EtOH–H_2_O (70:30 ratio) at 200 °C for 90 min in a reactor vessel, resulting in a lignin removal of 59.6% dm. To evaluate if the sample could be more delignified, two subsequent cycles in the same conditions were applied, reaching 67.4% dm in lignin removal (final value of 23.6 ± 0.2 g/100 g). However, differences in lignin removal between the second and third cycles were not significant, suggesting that delignification had reached its maximum extent, and if it is desired, subsequent bleachings may be performed. The first delignification cycle primarily extracts non-covalently bonded lignin, while the residual lignin probably undergoes progressive condensation, reinforcing ether linkages [239]. Similarly, de Hoyos-Martínez et al. [166] investigated the same sequential extraction but in almond shell biomass (initial composition of C: 18.2 g/100 g dm; H: 36.00 g/100 g dm; L: 31.2 g/100 g dm) under the same conditions. In the first cycle, a lignin removal of 78.0% dm was achieved, but as long as pre-treatments progressed, a final 87.7% dm of lignin removal was obtained after three cycles, being higher compared to that of walnut shells [177].

In both studies, cellulose content increased, however, de Hoyos-Martínez et al. [166] achieved a higher final cellulose yield after the three cycles (cellulose content of 48.6% dm) compared to Morales et al. [177] (cellulose content of 39.3% dm). To enhance cellulose recovery, an additional hydrothermal treatment at 210 °C was applied by Morales et al. [177], ultimately reaching a cellulose content of 48.3% dm. Jomnonkhaow et al. [240] also demonstrated the great combined effect of organosolv and hydrothermal pre-treatment together in Napier grass and silage.

A similar study investigated organosolv treatment for pistachio shells (initial composition of C: 57.5 g/100 g dm; H: 10.7 g/100 g dm; L: 29.1 g/100 g dm) under nearly identical conditions (EtOH–H_2_O 65:35) but incorporating MgSO_4_ as a catalyst [175]. The delignification rate was comparable to the previously mentioned studies, with both samples exhibiting approximately 10% less lignin content after pre-treatment (final lignin value of 19.7 g/100 g). Likewise, hemicellulose removal remained constant (value of 11.0 g/100 g), consistent with the values reported in the other investigations [166,177]. However, cellulose purification was lower in this case, achieving a 64.9 g/10 g content, likely due to the application of a single treatment cycle, whereas the other studies used three consecutive cycles, leading to a more extensive purification process. For that reason, different bleaching cycles were further applied to the matter, reaching up to an 87.4 g/100 g dm cellulose value after 1:10 *w*/*v* NaOH bleaching (pH = 12, 1 h at 98 °C) and another with a 3 M H_2_O_2_ solution (pH = 11, 2 h at 98 °C).

When comparing the results with a more conventional alkaline treatment, where bleaching steps were also applied (detailed in Section 7.1), a similar cellulose purification level was achieved for peanut shells as for hazelnut shells [140]. However, organosolv treatment is preferred due to its improved environmental sustainability, as it reduces the need for strong alkaline reagents and generates fewer by-products.

### 7.7. Microwave-Assisted Extraction (MAE) Pre-Treatment

Another promising green and sustainable pre-treatment method is microwave-assisted extraction (MAE) pre-treatment, which is increasingly considered a viable alternative to conventional chemical methods that often involve harsher environmental conditions and generate undesirable by-products [241].

The MAE method utilises electromagnetic waves to volumetrically heat the entire biomass matrix, simultaneously affecting both the surface and the core [242,243]. Unlike conventional heating methods—where heat transfer occurs through conduction, convection, or radiation from the exterior to the interior [244]—microwave irradiation directly transfers energy to the molecules. This energy absorption induces movement that generates dipole rotation and ionic conduction, leading to rapid and uniform heating. Since lignocellulosic biomass contains polar components, it efficiently absorbs microwave energy, making this method particularly viable for its processing [245]. As a result, reaction times are significantly reduced compared to other, more time-consuming, pre-treatment methods [246].

Usually, water is used as a solvent in MAE pre-treatment, which represents another advantage, as it eliminates the need for hazardous reagents. Additionally, water’s relatively high boiling point allows the process to reach temperatures suitable for efficient biomass fractionation. In particular, temperatures above 180 °C are critical, as this corresponds to the softening temperature of lignocellulosic biomass, at which the non-crystalline compound such as lignin matrix becomes more susceptible to microwaves and facilitates structural disruption of lignin and hemicellulose [247]. Also, microwave pre-treatment can replace conventional heating (e.g., hot plates) at any stage of cellulose extraction, providing more uniform and efficient heating when combined with solvents such as alkaline or acid solutions. This makes it a highly versatile approach for biomass fractionation in combination with pre-treatments such as alkaline [245] or with deep eutectic solvent [248].

Due to the limited research on this topic in nut shell biomass, the results obtained from different studies are scarce. In the investigation conducted by Harini and Chandra Mohan [176], a pre-treatment using water and microwave irradiation for 5 min across five cycles at 525 W was applied to walnut shells (initial composition of C: 42.4 g/100 g dm; H: 10.3 g/100 g dm; L: 27.2 g/100 g dm). However, the lignocellulosic composition at this stage was not reported and, instead, the results presented were after a subsequent 5% H_2_O_2_ bleaching for 30 min at 70 °C. This treatment resulted in a notable purification of cellulose, reaching up to 79.2 g/100 g, alongside almost complete removal of hemicellulose and a partial reduction in lignin.

Interestingly, the study by Özbek et al. [141] combined MAE pre-treatment with a 1.96 N NaOH solution, applying the process for only 158 s and 224 W to pistachio shells (initial composition of C: 42.4 g/100 g dm; H: 10.3 g/100 g dm; L: 27.2 g/100 g dm). In this case, a significant purification of cellulose was not achieved, although no degradation was observed, either. This suggests that microwave–alkaline treatment alone, and also in a single cycle, may not achieve high cellulose purity due to the lack of lignin removal, but its effectiveness could be improved with bleaching steps.

Microwave-assisted pre-treatment has also been combined with organosolv treatment [151], using ethanol (67%, 1:10 *w*/*v*) and a small amount of sulfuric acid as a catalyst. The process promotes lignocellulose breakdown by cleaving ether and ester lignin bonds and disrupting hydrogen bonds between hemicellulose and cellulose. Conducted at 150 °C for 30 min, this more intense treatment yielded 88.8% dm cellulose purity, demonstrating that higher processing intensity improves purification but may require more energy and possible degradation of polysaccharides.

### 7.8. Ultrasound Pre-Treatment

Ultrasound pre-treatment for nut shells is a technique that utilises high-intensity acoustic energy to enhance the breakdown of lignocellulosic biomass [249]. The mechanism seems to be based on acoustic cavitation, which generates localised high temperatures and pressures, leading to the disruption of cell wall structures and facilitating solvent penetration [250].

Despite its promising results, only a few studies are addressing its effectiveness in such recalcitrant biomasses. One notable study by El Khayat Driaa et al. [251] applied ultrasound treatment to walnut shells (initial composition of lignin of 34.9 ± 0.7 g/100 g dm), reporting interesting results. Using a titanium 14 mm ultrasound probe (in distilled water, 70 °C, 400 W), a notably enhanced hemicellulose extractability was observed, yielding a cellulose recovery of 43.46% dm, which was indicative of the method’s efficacy. These improvements were attributed to the cavitation effect, which disrupts the cellulose matrix and increases its accessible surface area.

Overall, the application of ultrasound pre-treatment to nut shells remains underexplored. However, some studies can be found in different lignocellulosic agricultural residues [252,253]. For instance, ultrasound-assisted cellulose extraction has been demonstrated in rice straw [254] (initial composition of C: 36.7 g/100 g dm; H: 19.3 g/100 g dm; L: 21.2 g/100 g dm), where ultrasound was applied as a preliminary step (40% amplitude, 750 W, 30 min) before bleaching treatment (1.7% NaClO_2_ solution, 4 h in reflux, three times). Results showed a cellulose and lignin final content of 65.9 and 5.2 g/100 g dm, respectively, thanks to the high amount of energy transferred to the sample, which disrupted the plant cell wall [255,256].

## 8. Conclusions

As discussed throughout this review, nut by-products are generated in substantial quantities worldwide, constituting a significant source of lignocellulosic biomass. Their production is intrinsically linked to geographical and climatic conditions, as well as agricultural practices and crop distribution, with certain regions experiencing notably higher accumulation rates. The continuous generation of these agro-industrial by-products poses an environmental challenge, as their disposal or incineration contributes to pollution.

In response, the valorisation of nut shells has attracted considerable attention in recent decades, with numerous studies investigating cellulose extraction using a variety of chemical and physical strategies. As highlighted throughout this review, innovative pre-treatment strategies and extraction methods have demonstrated promising results, underscoring their potential for sustainable biomass utilisation. However, the successful transition of these methods from laboratory-scale experimentation to industrial application demands rigorous validation under realistic operational conditions. This requires not only technical efficiency but also economic and energetic balances, which can only be achieved through an in-depth understanding of the raw material under different pre-treatment and treatment conditions.

Thus, ongoing research remains key for process optimisation, ensuring that these valorisation strategies are both scalable and environmentally friendly. Future efforts must prioritise the exploration of less conventional, emerging techniques—such as deep eutectic solvents, ultrasound, or hydrothermal—over traditional, often hazardous chemical routes. Continued innovation and optimisation of biomass processing strategies are essential to enable the effective exploitation of lignocellulosic biomass, supporting the transition toward more sustainable industrial practices and a circular economy.

## Figures and Tables

**Table 2:** Comparative summary of methods for cellulose isolation from nut shells by-products.

				Cellulose (g/100 g dm)		Hemicellulose (g/100 g dm)		Lignin (g/100 g dm)		Ref.
Nut Type	Area	Method	Conditions	Untreated Matter	Treated Matter	Untreated Matter	Treated Matter	Untreated Matter	Treated Matter	
Almond	Guara	Hydrothermal	30 min, 180 °C	26.8 ± 1.3	41.7 ± 0.9	23.6 ± 0.2	11.6 ± 0.1	21.2 ± 2.0	18.6 ± 0.1	[40]
		Hydrothermal + bleaching	30 min, 180 °C bleaching 8% H_2_O_2_ pH = 12, 1 h (×4)	26.8 ± 1.3	78.4 ± 0.2	23.6 ± 0.2	12.2 ± 1.2	21.2 ± 2.0	4.9 ± 1.2	
		Hydrothermal + bleaching	30 min, 180 °C, bleaching 1.7% NaClO_2_, 4 h (×7)	26.8 ± 1.3	83.7 ± 2.4	23.6 ± 0.2	12.5 ± 0.7	21.2 ± 2.0	5.1 ± 0.6	
	Marcona	Organosolv	EtOH:H_2_O (70:30), 90 min 200 °C (×3)	18.19 ± 0.19	48.63 ± 1.27	35.99 ± 1.23	22.15 ± 0.26	31.24 ± 0.29	14.85 ± 0.40	[166]
Chestnut	Spain	Hydrothermal	non-isothermal regimen, 180 °C	20.6 ± 1.4	26.0 ± 1.6	10.5 ± 0.5	6.4 ± 0.1	44.6 ± 1.4	61.2 ± 1.6	[167]
Hazelnut	Levante	Hydrothermal	biomass:H_2_O 1:10, 0.5 h, 180 °C	18.7 ± 0.5	27.8 ± 0.9	18.7 ± 0.1	4.5 ± 0.5	46.7 ± 0.2	58.7 ± 0.3	[153]
		Hydrothermal	biomass H_2_O 1:10, 0.5 h, 190 °C	18.7 ± 0.5	30.4 ± 1.1	18.7 ± 0.1	2.2 ± 0.2	46.7 ± 0.2	63.1 ± 1.5	
		Alkaline	2.25% NaOH, 0.5 h, 60 °C	16.7	18.1	13.3	12.4	51.3	32.8	[168]
		Dilute acid	0.5% H_2_SO_4_, 0.5 h, 120 °C	16.7	21.6	13.3	7.8	51.3	60.0	
		Hydrothermal	biomass:H_2_O 1:10, 0.5 h, 120 °C	16.7	23.2	13.3	9.2	51.3	63.5	
		Hydrothermal + dilute acid	H_2_O, 0.5 h, 120 °C 0.5% H_2_SO_4_, 0.5 h, 120 °C	16.7	29.5	16.7	2.2	51.3	67.0	
		Dilute acid	1% H_2_SO_4_, 0.25 h, 120 °C	16.67	21.25	13.30	5.90	51.30	45.14	[169]
		Alkaline	2.25% NaOH, 1 h, 150 °C	16.67	12.07	13.30	4.73	51.30	28.80	
		Hydrothermal	H_2_O, 0.25 h	16.67	16.29	13.30	2.03	51.30	12.35	
	Gilan	Alkaline + bleaching	3% NaOH, pH = 12, 3 h, 80 °C bleaching 2.7% NaClO_2_, pH = 5, 1 h, 80 °C (×3)	32.1 ± 2.0	70.8 ± 1.3	17.9 ± 2.4	8.6 ± 0.9	38.7 ± 1.1	11.5 ± 3.2	[170]
	Spain	Alkaline organosolv	4% NaOH:EtOH (50:50), 1 h, 135 °C	24.2 ± 0.1	29.3 ± 0.5	23.2 ± 0.1	19.3 ± 0.8	39.7 ± 0.61	42.6 ± 3.5	[171]
		Hydrothermal + alkaline organosolv	15 min, 60 °C to 210 °C 4% NaOH:EtOH (50:50), 1 h, 135 °C	24.2 ± 0.1	42.8 ± 0.7	23.2 ± 0.1	3.6 ± 0.1	39.7 ± 0.61	48.9 ± 2.5	
		Acid organosolv	H_2_SO_4_:EtOH (60:40), 1 h, 180 °C	24.2 ± 0.1	54.0 ± 1.2	23.2 ± 0.1	11.9 ± 0.9	39.7 ± 0.61	29.6 ± 0.7	
		Hydrothermal + acid organosolv	15 min, 60 °C to 210 °C H_2_SO_4_:EtOH (60:40), 1 h, 180 °C	24.2 ± 0.1	55.4 ± 1.9	23.2 ± 0.1	1.7 ± 0.3	39.7 ± 0.61	40.5 ± 1.2	
Macadamia	Yunnan	DESs + bleaching	ChCl:OA (1:1), 2 h, 110 °C bleaching 10% NaClO_2_, 2 h, 80 °C	28.25 ± 1.02	not reported	16.74 ± 0.61	not reported	34.11 ± 1.15	not reported	[152]
	Yunnan	DESs + bleaching	K_2_CO_3_:glycerol (1:7), 2 h, 110 °C bleaching 10% NaClO_2_, 2 h, 80 °C	28.25 ± 1.02	not reported	16.74 ± 0.61	not reported	34.11 ± 1.15	not reported	
	Beaumont	Dilute acid	0.1 M HCl, 3 h	34.48	29.54	21.48	24.31	11.91	31.90	[19]
		Alkaline	0.1 M NaOH, 3 h	34.48	40.64	21.48	17.47	11.91	31.68	
	NSW	Ionic liquids	[Emim][OAc], 18 h, 110 °C	not reported	not reported	not reported	not reported	35.50	29.20	[172]
Peanut	China	Alkaline + bleaching	2% NaOH, 24 h bleaching 8% H_2_O_2_, 8 h, 50 °C bleaching 2% NaClO_2_, 4 h, 75 °C	45.3 ± 1.5	79.7 ± 0.7	8.84 ± 1.2	12.3 ± 0.8	30.3 ± 0.61	4.2 ± 0.3	[140]
	Jiangsu	DESs + derived lignin phenol	ChCl:guaiacol (1:1), 3 h, 120 °C	36.39 ± 0.71	37.47 ± 1.00	15.64 ± 0.50	15.28 ± 0.77	25.01 ± 0.94	23.30 ± 0.17	[173]
		DESs + derived lignin phenol	ChCl:guaiacol:AlCl_3_ (1:1:0.01), 3 h, 120 °C	36.39 ± 0.71	49.03 ± 0.78	15.64 ± 0.50	8.57 ± 0.88	25.01 ± 0.94	17.80 ± 0.11	
		DESs + derived lignin phenol	ChCl:guaiacol:AlCl_3_ (1:1:0.07), 3 h, 120 °C	36.39 ± 0.71	59.49 ± 0.78	15.64 ± 0.50	1.98 ± 0.11	25.01 ± 0.94	12.86 ± 0.44	
Pistachio	Fandoghi	Alkaline + bleaching	2% NaOH, 4 h, 100 °C bleaching 1.7% NaClO_2_, 6 h, 80 °C	38.1 ± 1.9	71.3 ± 3.4	31.4 ± 2.7	17.0 ± 3.4	23.6 ± 3.0	8.2 ± 1.5	[174]
	Turkey	Microwave alkali	1.96 N NaOH, 2.63 min, 142 °C, 224 W	25.15 ± 0.88	40.40	35.04 ± 1.12	31.31	25.95 ± 1.30	26.86	[141]
	Commercial	Organosolv	EtOH:H_2_O (65:35), 0.05 M MgSO_4_	57.54	64.98	10.73	10.96	29.11	19.67	[175]
		Organosolv + bleaching	EtOH:H_2_O (65:35), 0.05 M MgSO_4_ NaOH 1:10, pH = 12, 1 h, 98 °C	57.54	85.39	10.73	10.53	29.11	3.37	
		Organosolv + bleaching	EtOH:H_2_O (65:35), 0.05 M MgSO_4_ NaOH1:10, pH = 12, 1 h, 98 °C 3 M H_2_O_2_, pH = 11, NaOH/ Mg(OH)_2_ (3:1), 5 mmol GLDA, 2 h, 98 °C	57.54	87.38	10.73	12.62	29.11	0	
	Kerman	Microwave organosolv	67% EtOH, 0.5 h, 150 °C	31.2 ± 2.2	88.8 ± 3.2	31.3 ± 1.3	4	21.2 ± 1.5	5 ± 2	[151]
Walnut	India	Microwave + bleaching	5 min, 525 W (×5) 5% H_2_O_2_, 0.5 h, 70 °C	42.36 ± 2.11	79.24 ± 2.51	10.26 ± 1.16	0.28 ± 0.10	27.19 ± 2.43	6.18 ± 1.24	[176]
	Henan	DESs	ChCl:TsOH:EG (1:1:1), 2 h, 110 °C	33.35 ± 0.83	83.54 ± 0.72	22.90 ± 0.66	3.83 ± 0.36	38.56 ± 0.63	9.38 ± 0.35	[154]
			ChCl:LA:EG (1:1:1), 4 h, 120 °C	33.35 ± 0.83	47.87 ± 1.02	22.90 ± 0.66	13.02 ± 1.03	38.56 ± 0.63	33.34 ± 0.64	
			ChCl:OA:EG (1:1:1), 4 h, 120 °C	33.35 ± 0.83	60.51 ± 0.94	22.90 ± 0.66	9.54 ± 0.41	38.56 ± 0.63	26.23 ± 1.22	
			ChCl:CA:EG (1:1:1), 4 h, 120 °C	33.35 ± 0.83	44.68 ± 1.80	22.90 ± 0.66	15.48 ± 1.32	38.56 ± 0.63	36.20 ± 1.15	
	Gipuzkoa	Organosolv	EtOH:H_2_O (70:30), 90 min, 200 °C (×3)	18.75	39.3 ± 1.21	27.65	16.17 ± 0.44	33.25	23.59 ± 0.16	[177]
		Organosolv + hydrothermal	EtOH:H_2_O (70:30), 90 min, 200 °C (×3) 210 °C	18.75	48.32 ± 0.39	27.65	5.41 ± 0.58	33.25	23.02 ± 2.71	
	China	DESs	ChCl:TsOH:EG (1:1:2), 2.5 h, 90 °C	32.87 ± 0.95	74.79 ± 1.58	22.53 ± 0.76	4.76 ± 0.72	38.56 ± 0.69	12.88 ± 0.83	[178]
	Shanxi	Alkaline	2 wt% NaOH, 4 h, 100 °C (×4)	27.4	56.6	31.3	7.6	36.31	30.98	[179]
		Alkaline + bleaching	2 wt% NaOH, 4 h, 100 °C (×4) bleaching 1.7 wt% NaClO_2_, 6 h, 80 °C	27.4	87.9	31.3	1.8	36.31	0.17	[180]

**Abbreviations: TsOH**: p-toluenesulfonic acid; **GLDA**: N,N-dicarboxymethyl glutamic acid tetrasodium salt.

## Data Availability

Data presented in this review was obtained from online sources, including both open-access and subscription publications.

## Abbreviations

The following abbreviations are used in this manuscript:

AcHBD	Acidic hydrogen bond donor	[Emim] [CH_3_CO_2_]	1-Ethyl-3-methylimidazolium acetate
AIBP	Agro-industrial by-product	HBA	Hydrogen bond acceptor
AP	Alkaline pre-treatment	HBD	Hydrogen bond donor
APD	Alkaline peroxide delignification	IL	Ionic liquid
AS	Almond shell	IOD	Ionic solvent delignification
[Bmim][Cl]	1-Butyl-3-methylimidazolium hexafluorophosphate chloride	LA	Lactic acid
CA	Citric acid	LHW	Liquid hot water (hydrothermal)
ChCl	Choline chloride	MAE	Microwave-assisted extraction
CNSL	Cashew nut shell liquid	MT	Metric tons
DAP	Dilute acid pre-treatment	SWE	Subwater critical extraction
DES	Deep eutectic solvent	OA	Oxalic acid
DESD	DES pre-treatment	*v/v*	Volume/volume percentage (%)
EG	Ethylene glycol		
